# Fast Classification of Geographical Origins of Honey Based on Laser-Induced Breakdown Spectroscopy and Multivariate Analysis

**DOI:** 10.3390/s20071878

**Published:** 2020-03-28

**Authors:** Zhangfeng Zhao, Lun Chen, Fei Liu, Fei Zhou, Jiyu Peng, Minghua Sun

**Affiliations:** 1 Key Laboratory of E & M, Zhejiang University of Technology, Ministry of Education & Zhejiang Province, Hangzhou 310014, China; i12fly@163.com (Z.Z.); 15857185317@163.com (L.C.); jypeng@zjut.edu.cn (J.P.); 2 College of Biosystems Engineering and Food Science, Zhejiang University, Hangzhou 310058, China; fliu@zju.edu.cn; 3 College of Standardization, China Jiliang University, Hangzhou 310018, China; 4 Hangzhou Landa Science and Technology Co., Ltd., Hangzhou 310030, China; landast@126.com

**Keywords:** honey, geographical origin, multivariate analysis, classification, laser-induced breakdown spectroscopy

## Abstract

Traceability of honey is highly required by consumers and food administration with the consideration of food safety and quality. In this study, a technique named laser-induced breakdown spectroscopy (LIBS) was used to fast trace geographical origins of acacia honey and multi-floral honey. LIBS emissions from elements of Mg, Ca, Na, and K had significant differences among different geographical origins. The clusters of honey from different geographical origins were visualized with principal component analysis. In addition, support vector machine (SVM) and linear discrimination analysis (LDA) were used to quantitively classify the origins. The results indicated that SVM performed better than LDA, and the discriminant results of multi-floral honey were better than acacia honey. The accuracy and mean average precision for multi-floral honey were 99.7% and 99.7%, respectively. This study provided a fast approach for geographical origin classification, and might be helpful for food traceability.

## 1. Introduction

Honey is a natural sweet product produced by bees from the nectar of flowers [1], which mainly consists of carbohydrates, water, proteins, minerals, amino acids, phenols, and vitamins, etc. Because of its high nutrients and healthy benefits, honey has been considered as an important health product around the world, especially in China. It has been demonstrated that honey can improve immune systems and oral health, prevent side effects linked with cancers treatment, heal wounds, etc. [2,3]. However, the constitutes of honey has regional features, and it is likely influenced by the climate, altitude, and other environmental factors [4,5]. Hence, the supplemental information concerning the geographical origin should be given according to Food Safety Law of the People’s Republic of China.

In order to determine the geographical origins of honey, some analytical methods have been proposed by researchers. Chemical analysis methods including high performance liquid chromatography–mass spectrometry/mass spectrometry [6], isotope ratio mass spectrometry [7], inductively coupled plasma optical emission spectroscopy [8,9,10], and gas chromatography mass spectrometry [9] were used to discriminate the geographical and botanical origins of honey. Regional and botanical differences in chemical substances contribute to the discrimination. However, the sample preparation of these methods is time-consuming, and lots of reagents are needed. Recently, some fast detection methods were also utilized to classify honey origins, which including Terahertz time-domain attenuated total reflection spectroscopy [11], electronic tongue [12,13], electronic nose [13,14], infrared spectroscopy [13,14], Raman spectroscopy [14,15], etc. These methods might provide novel approaches for in-line detection, whereas further study is still needed to improve the detection accuracy and stability.

Laser-induced breakdown spectroscopy (LIBS) is a laser-based spectroscopy, which can obtain the *fingerprint* information of samples by analyzing the emission spectra. Because of the advantages of fast detection, environment-friendly feature, and multi-element analytical capability, LIBS has gained continuous attention in industrial [16], environmental [17], and food safety applications [18]. Based on the regional differences in elemental concentration, the geographical origins of honey might be differentiated by LIBS. As so far, no relevant study concerning the application of LIBS for discriminating honey geographical origins has been published. In addition, multivariate methods have been proven as an effective tool in extracting valuable information from raw data and establishing models for discrimination. A review concerning the application of multivariate methods for prediction of botanical and geographical origin of honey has been recently published [19].

Hence, LIBS combined with multivariate methods were used to discriminate the geographical origins of honey. The specific aims of this study are (1) to analyze the LIBS spectral features of different geographical origins of honey; (2) to reduce the data dimension and determine feature variables that contributing regional difference; (3) to establish models for classification of honey origins based on multivariate methods.

## 2. Materials and Methods

### 2.1. Sample Preparation

Honey from different geographical origins were collected from local producers. According to the varieties of nectar of flowers, honey can be divided into uni-floral honey and multi-floral honey. And acacia honey is one of high-valuable and representative uni-floral honeys. Hence, two different honey categories (acacia honey and multi-floral honey) were used in this experiment. Each honey category had three different geographical origins, and the sample number for each group is 40. The general information of honey samples is listed in Table 1.

### 2.2. LIBS Measurement

Before LIBS analysis, the honey samples (8 g) were added in 12-well plates. No other sample preparation was needed. A laboratory-assembled LIBS device was used to analyze samples, the detailed of which has been introduced in previous research [20]. In this experiment, a laser (Vlite 200, Beamtech, Beijing, China) was used to ablate samples at the second-harmonics wavelength (532 nm), with ablation energy of 80 mJ and frequency of 1 Hz. The focal length of lens is 100 mm, and the lens-to-sample-distance (LTSD) in this case was 99 mm. The plasma light was collected by a UV-NIR achromatic mirror system (CC52, Andor, Belfast, UK), and transferred to an Echelle spectrograph (ME 5000, Andor, Belfast, UK), finally detected by an intensified charge coupled device (ICCD, DH334T-18F-03, Andor, Belfast, UK). The delay time, integration time, and relative gain of ICCD were 2 µs, 10 µs, and 26, respectively. Before experiment, the intensity of ICCD was calibrated by a deuterium tungsten halogen source (DH-2000-BAL-CAL, Ocean Optics, Largo, FL, USA), and the wavelength of spectrograph was calibrated with a mercury argon lamp (HG-1, Ocean Optics, Largo, FL, USA). LIBS measurement was performed by single shot scanning in an ablation region of 10 × 10 mm with a resolution of 1 mm. Hence, 100 successive shots were performed for each sample, and 100 spectra were collected.

### 2.3. Multivariate Analysis

All spectra from the same origin were used for representing regional characteristics. A total of 4000 spectra were obtained for each origin. In order to establish and verify discriminant model, thirty samples (3000 spectra) were randomly assigned to a calibration set, and the rest (1000 spectra) were in the prediction set. In this study, principal component analysis (PCA) was used to quantitively visualize the distribution of honey (the calibration samples) with score plots, and linear discriminant analysis (LDA) and support vector machine (SVM) were used for quantitatively classifying geographical origins. PCA, LDA, and SVM analysis was done in the MATLAB (v2018, The MathWorks Inc., Natick, MA, USA).

PCA is an unsupervised cluster algorithm which reduces data dimensions through projecting variables into some principal components (PCs) with maximal variations [21]. It can serve as a useful first step before classification of samples [22]. Because the number of original variables was large (more than 20,000), and lots of them were redundant variables. Hence, the first few principal components could be used to visualize sample distribution in score plots and represent the majority of spectral information. In addition, the loadings represent the contributions to PCs, which could be used to determine feature variables. In PCA model, LIBS spectra were used as inputs.

LDA and SVM were two popular multivariate analysis algorithms, both of which has been widely used in solving classification problems [23,24]. LDA is a supervised classification algorithm based on Bayes’ formula, which linearly transforms the samples into a lower dimensional space, so that the samples belong to the same class cluster together [25]. The objective of LDA is to determine the best fit parameters for classification. It is simply to carry out and can be computed fast enough for in-line application. Hence, spectral sensors combined with LDA has been widely applied in food quality control [26,27], and produce good results. However, because of the strong dependence of assumption in its derivation, factors such as noise, non-Gaussian data distribution, and outliers might have a detrimental effect on LDA’s performance [28]. Hence, SVM that performed good in discrimination was also used to classify the geographical origins of honey.

SVM is a supervised non-parametric statistical learning algorithm, which has been used for solving complex separations [23]. There is no assumption made on the data distribution. First, kernel function was used to map the data into a higher dimensional feature space which is separable with linear algorithms. Then, a hyperplane with maximum margin was determined to separate different classes. In order to solve multi-class separations in this case, one-against-one multiclass method was used.

In contrast to PCA, the dependent variables (group labels) are also considered in LDA and SVM when modeling. In this case, the independent variables (X) were the first few PCs, the dependent variables are the group labels of geographical origins. Moreover, 10-folds cross validation were used to avoid overfitting.

In addition, confusion matrix, accuracy, mean average precision (MAP), precision, and recall of each model were used to evaluate model quality. Confusion matrix is a commonly used tool representing classification results. On a confusion matrix, the row corresponds to the output class, and the column corresponds to the target class. Each cell represents the number of samples belongs to target class whereas classified as predicted class. Hence, the diagonal cells correspond to observations that are correctly classified. The off-diagonal cells correspond to incorrectly classified observations. Other figures of merit including accuracy, MAP, precision, and recall could be calculated from confusion matrix. Accuracy and MAP measure average performance of multiclass results, whereas precision and recall correspond to the performance for each class. Accuracy is the measure of the true results. Precision measures the correctly classified number in each output class. Recall measures the correctly classified number in each target class. These figures of merit present values in the range from 0 to 1. The values of being 1 for accuracy, MAP, precision, and recall indicate the best model. The equations were as follows:

Precision = true positives/number of positive(1)

Recall = true positive/(true positive + false negative)(2)

Accuracy = (true positive + true negative)/(true positive + true negative + false positive + false negative)(3)

### 2.4. One-Way ANOVA Test

In order to examine the regional difference of LIBS emissions, one-way ANOVA test was performed. In this case, 33 of peak intensity of main emissions was considered as dependent variables, and the class label was considered as independent variable. The main emission lines can be identified according to National Institute of Standards and Technology (NIST) database [29]. Values were reported as the mean ± standard deviation (SD), and one-way ANOVA test was performed by SPSS (ver. 25.0, SPSS Inc., Chicago, IL, USA). Duncan’s test was used to determine the significance level (*p* < 0.05).

## 3. Results and Discussion

### 3.1. Spectral Characteristics of Honey

**Figure 1:**
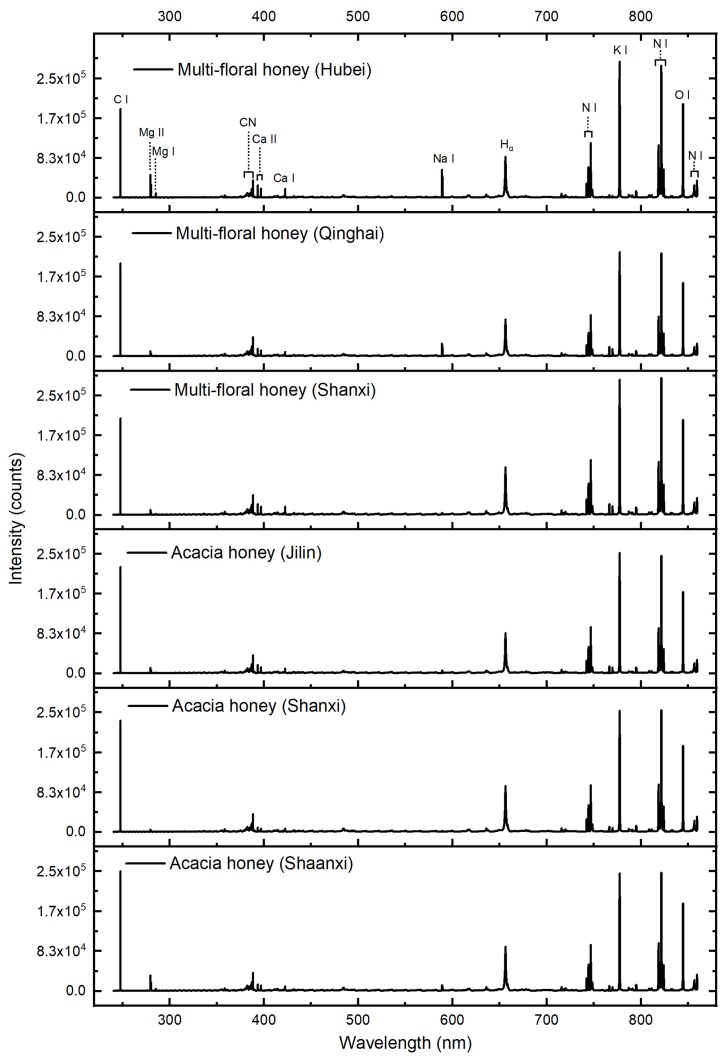
Spectral fingerprints of honeys from different geographical origins.

The LIBS spectra offer fingerprint data of honey that contains the regional information. Because of the climate, temperature, and environmental factors, honey from different geographical origins might have different elemental constitutes [4]. The elemental difference could be visualized through LIBS spectra. Figure 1 shows the LIBS average spectra of honey from different origins. Two categories (acacia honey and multi-floral honey) from three different origins were analyzed. Each peak wavelength in LIBS spectrum represented the specific element that could be identified in NIST database, and the peak intensity was related to elemental concentration. As shown in Figure 1, the tendency of LIBS spectrum from different origins was similar. However, slight difference in peak intensity of different honey origins could be found. The emissions (Mg II 279.55, Mg II 280.27, and Mg I 285.21 nm) from A1 (acacia honey, Shaanxi) and M3 (multi-floral honey, Hubei) were significantly stronger than those from other origins. In addition, the emissions (Na I 589.00 and Na I 589.59 nm) from A1 (acacia honey, Shaanxi) were stronger than those from other origins, which indicated high Na concentration in multi-floral honey from Hebei. Other differences such as emissions of Ca I 422.67, K I 766.49, and K I 769.90 nm could also be observed. Due to the variation of constitutes in single group, it was hard to distinguish the origins with above mentioned rules. Hence, multivariate methods were further used to visualize the clusters and discriminate the geographical origins.

Table 2 shows the peak intensity of main emissions of honey. One-way ANOVA test was performed for six different groups. The emission marked in bold showed that the peak intensity had significant difference among at least five groups. It indicated that the emissions of Mg I 285.21, Ca II 393.37, Na I 589.00, Na I 589.59, K 766.49, and K I 766.90 nm might have distinguished differences among the five groups, which played an important role in discrimination. Moreover, the peak intensity of emission of Na I 589.00 nm has significant difference among the six groups. It might be considered as a feature emission for geographical and varietal classification. The significant differences of these emissions might provide fundamental signatures for the multivariate classification of honey origins.

### 3.2. PCA Analysis

**Figure 2:**
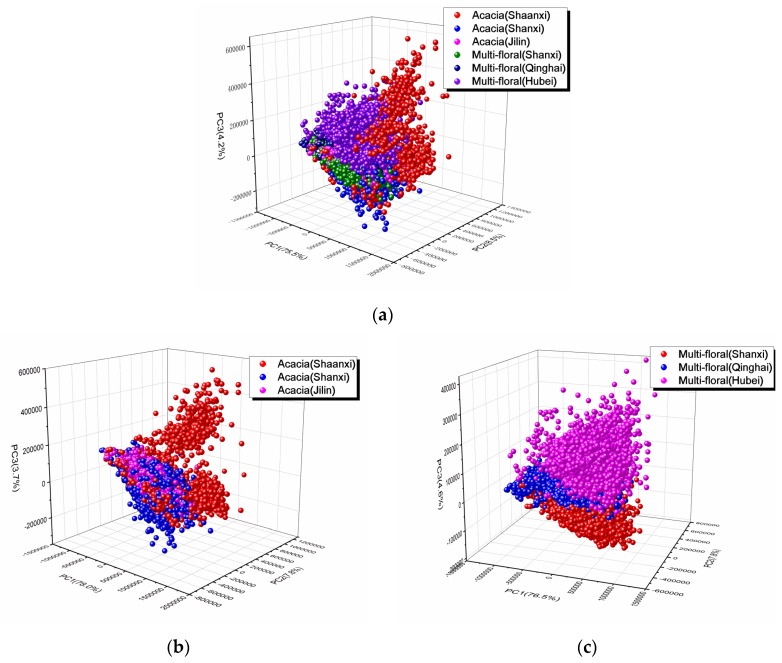
Principal component analysis (PCA) scatter plots for (**a**) all honey (including acacia honey and multi-floral honey), (**b**) acacia honey, and (**c**) multi-floral honey.

PCA analysis was used to visualize the clusters with scores plots, and determine the important variables with loadings plots. First, all honey (including acacia honey and multi-floral honey) within different geographical origins were visualized through PCA analysis. The contribution of the first three principal components accounted for 88.2% of explained variance, with PC1, PC2, and PC3 of 75.5%, 8.5%, and 4.2%, respectively. Figure 2a shows score plots of six different groups. In general, six groups entangled with each other. It might be credited to complex reciprocal effect of botanical and geographical origins. It was hard to distinguish with PCA analysis.

Therefore, PCA analysis was separately performed for acacia honey and multi-floral honey, the score plots of which are shown in Figure 2b,c. The contribution of the first three principal components for acacia honey and multi-floral honey accounted for 89.5% and 88.9% of the explained variance, respectively. Apparently, the classification result of multi-floral honey was better than that of acacia honey. The samples from Shanxi, Qinghai, and Hubei provinces clustered more compact, and could be separated.

**Figure 3:**
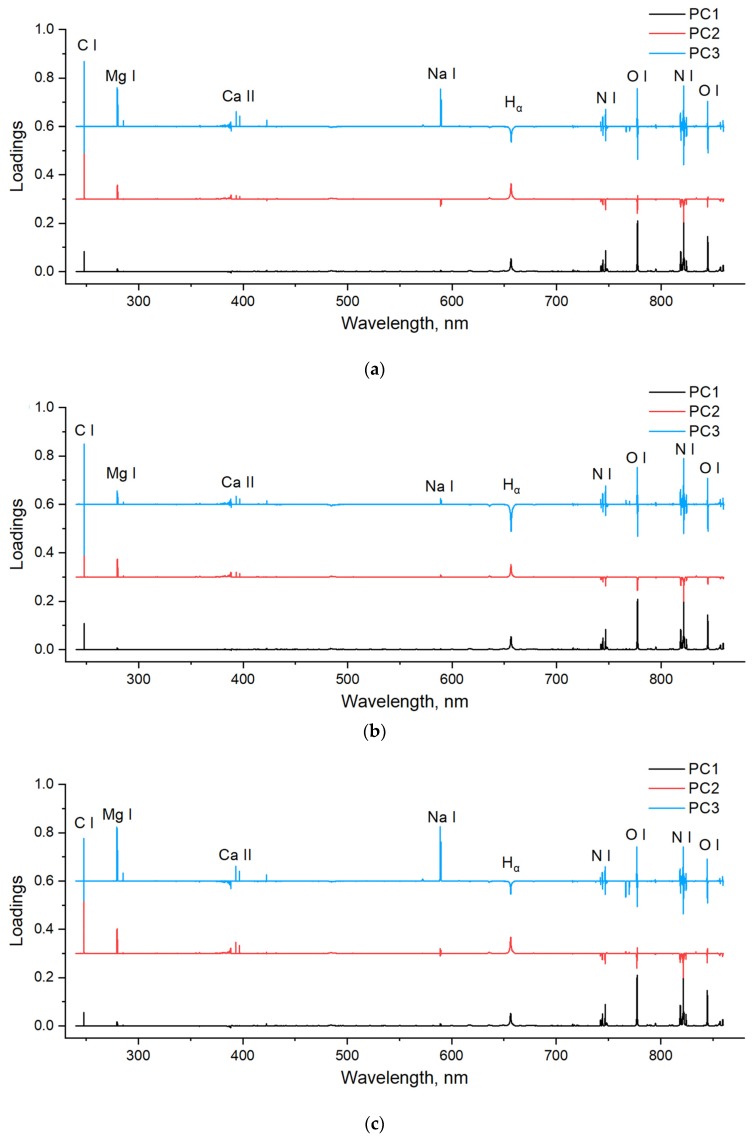
Loadings of the first three principal components for (**a**) all honey (including acacia honey and multi-floral honey), (**b**) acacia honey, and (**c**) multi-floral honey.

The loadings of PCA indicate the contribution of each variable, which can be used to determine feature variables. The larger absolute value of the loading, the more importance of the variable. In addition, positive value indicates a positive link, and negative value indicates a negative link. Because the first three principal components contributed most of the total variance (>85%), their loadings were used to determine important variables. Figure 3 shows loading plots of the first three principal components for all honey, acacia honey, and multi-floral honey. Similar trends could be observed for these three plots, and the variables with large absolute loadings corresponded to the main emissions. Most of loadings of PC1 (except spectral range of CN emissions) is positive, which indicated that there is a positive link between the variable and the information contained in PC1. As shown in Figure 3, the major elements of C, H, O, and N contributed to the discrimination, as well as the mineral elements of Mg, Ca, Na, and K.

### 3.3. Quantitative Discrimination

Because the variables of full spectrum were over 20,000, it might lead to overfitting and worsen calculation speed [30]. In this case, principal components after dimensional reducing were used to construct models. The first few principal components with accumulated variance over 95% were used to represent the raw variables. The number of PCs for all honey, acacia honey, and multi-floral honey were 26, 23, and 29, respectively. Then, these variables were used as the inputs of LDA and SVM models.

**Figure 4:**
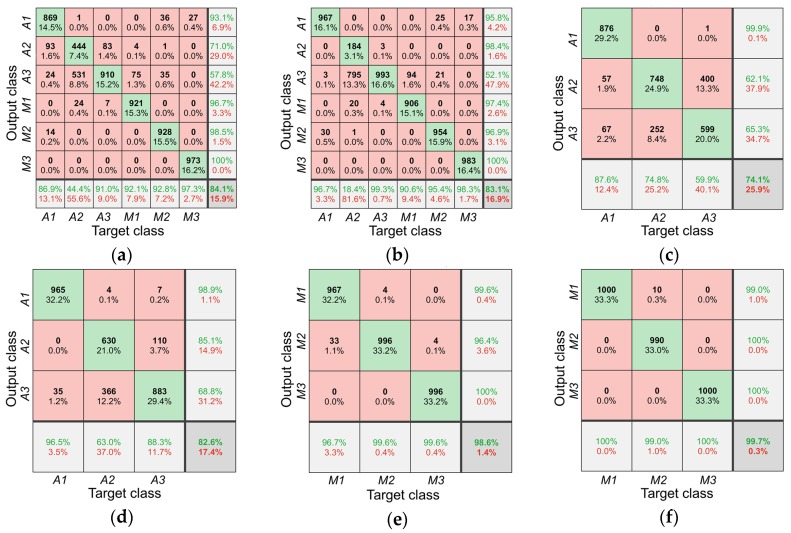
Confusion matrix for origin discrimination of all honey (**a**) LDA model and (**b**) SVM model, acacia honey (**c**) LDA model and (**d**) SVM model, multi-floral honey (**e**) LDA model and (**f**) SVM model. The diagonal cells correspond to observations that are correctly classified. The off-diagonal cells correspond to incorrectly classified observations. Both the number of observations and the percentage of the total number of observations are shown in each cell. The column on the far right of the plot shows the percentages of all the examples predicted to belong to each class that are correctly and incorrectly classified. These metrics are often called the precision and false discovery rate, respectively. The row at the bottom of the plot shows the percentages of all the examples belonging to each class that are correctly and incorrectly classified. These metrics are often called the recall and false negative rate, respectively. The cell in the bottom right of the plot shows the overall accuracy.

Confusion matrix was used to evaluate the performance (Figure 4). For all honey, the accuracy of LDA and SVM models were 84.1% and 83.1%, respectively. In LDA model, 93 acacia honey samples from Shaanxi province was misclassified as Shanxi province, and 531 acacia honey samples from Shanxi province were misclassified as those from Jilin province. In SVM model, the largest misclassification was from the samples from Shanxi province; 795 acacia honey samples from Shanxi province was misclassified as those from Jilin province. The results indicated that it was hard to discriminate the samples from Shanxi province and Jilin province. For acacia honey, the accuracy of LDA and SVM models were 74.1% and 82.6%, respectively. The low accuracy was also originated from the misclassification between Shanxi province and Jilin province. The recall of Shanxi province and Jilin province in LDA model were 74.8% and 59.9%, whereas 63.0% and 88.3% in the SVM model. For multi-floral honey, the accuracy of the LDA and SVM models were 98.6% and 99.7%, respectively. In the LDA model, only 33 samples from Shanxi province were misclassified as those from Qinghai Province. The performance of the SVM model was better than the LDA model.

In addition, a comparison of modeling performance in three classifications is listed in Table 3. Accuracy and mean average precision were used to evaluate the performance. In general, the SVM model performed better than the LDA model. The accuracy for all honey, acacia honey, and multi-floral honey were 83.1%, 82.6%, and 99.7%, and the mean average precision were 79.3%, 89.5%, and 99.7%. The discrimination performance of all honey was worse than acacia honey or multi-floral honey. The geographical origin of multi-floral honey was well classified, with accuracy and mean average precision of 99.7% and 99.7%. Great differences have been found among these three origins, which were mainly caused by the botanical difference. The flowers in Qinghai Province were mainly rhodiola rosea, chrysanthemum, codonopsis pilosula, and hippophae rhamnoides, etc. The flowers in Shanxi Province were mainly chaste, jujube, and acacia, etc. The flowers in Hebei Province were mainly Chinese medical plants, such as goldthread, chrysanthemum, etc. The constituents of honey might be affected by the regional difference of botanical variety.

## 4. Conclusions

Laser-induced breakdown spectroscopy was successfully used to discriminate the geographical origins of honey. Spectral intensity of emissions from Mg, K, Ca, and Na showed slight difference among different origins. One-way ANOVA test indicated emissions from Na I 589.00 nm had significant difference among six groups, which might be considered as feature emission for geographical origin discrimination. Different clusters of origins in multi-floral honey could be separated in PCA score plot, whereas the samples from all honey (including acacia honey and multi-floral honey) and acacia honey were entangled with each other. Emissions from major elements C, H, O, and N as well as Mg, Ca, Na, and K had large loading values, which indicated the importance in each principal component. In addition, the geographical origins of all honey, acacia honey, and multi-floral honey were quantitatively discriminated with LDA and SVM. In general, the SVM model performed better than the LDA model. For acacia honey, the accuracy and mean average precision were 82.6% and 89.5%. Some deep learning methods such as convolutional neural networks might be used to further improve the performance. Excellent discriminant result was achieved in multi-floral honey, with accuracy and mean average precision of 99.7% and 99.7%, respectively. It might be credited to the regional difference in botanical variety. The results indicated the feasibility of the utilization of LIBS for discriminating the geographical origins of honey, which might provide an approach for food traceability.

## Figures and Tables

**Table 1:** General information of honey samples.

Variety	Sample Code	Origin	No. of Samples
Acacia honey	A1	Shaanxi	40
	A2	Shanxi	40
	A3	Jilin	40
Multi-floral honey	M1	Shanxi	40
	M2	Qinghai	40
	M3	Hubei	40

**Table 2:** Peak intensity of the main emissions from honey.

No.	Observed Wavelength (nm)	Ritz Wavelength (nm)	Emissions	Peak Intensity (×10^3^, Counts) *					
				A1	A2	A3	M1	M2	M3
1	247.88	247.86	C I	255.28 ± 78.77^a^	235.05 ± 30.74^a,b^	224.46 ± 25.66^b,c^	203.69 ± 36.67^c,d^	195.31 ± 42.15^d^	190.38 ± 59.54^d^
2	279.58	279.55	Mg II	33.68 ± 11.60^a^	5.27 ± 1.09^b^	11.92 ± 2.89^c^	10.81 ± 3.83^c^	11.33 ± 4.07^c^	50.35 ± 26.26^d^
3	280.28	280.27	Mg II	17.90 ± 6.32^a^	3.03 ± 0.61^b^	6.40 ± 1.62^c^	6.04 ± 2.18^c^	6.28 ± 2.15^c^	29.26 ± 115.02^d^
**4**	**285.23**	**285.21**	**Mg I**	**5.14 ± 1.39^a^**	**1.26 ± 0.19^b^**	**2.10 ± 0.48^c^**	**2.97 ± 0.63^d^**	**2.27 ± 0.30^c^**	**10.27 ± 1.79^e^**
5	385.07	385.01	CN 4-4	10.49 ± 1.50^a^	10.28 ± 0.92^a^	10.27 ± 0.95^a^	11.71 ± 0.96^b^	10.99 ± 0.97^c^	10.32 ± 0.75^a^
6	385.47	385.44	CN 3-3	10.34 ± 1.47^a,b^	10.04 ± 0.88^a^	9.91 ± 0.94^a^	11.19 ± 0.91^c^	10.55 ± 0.99^b^	10.14 ± 0.64^a,b^
7	386.19	386.15	CN 2-2	12.87 ± 1.59^a,b^	12.68 ± 1.05^a,b^	13.18 ± 1.21^b^	15.00 ± 1.27^c^	14.11 ± 1.29^d^	12.38 ± 1.44^a^
8	387.13	387.12	CN 1-1	19.5 ± 2.78^a,b^	19.10 ± 1.61^a^	19.71 ± 2.05^a,b^	22.07 ± 1.81^c^	21.19 ± 2.18^c^	20.07 ±1.40^d^
9	388.33	388.32	CN 0-0	38.34 ± 4.92^a^	37.70 ± 3.21^a^	38.10 ± 3.99^a^	41.88 ± 3.60^b^	40.24 ± 4.18^b^	37.22 ± 3.06^a^
**10**	**393.37**	393.37	**Ca II**	**14.73 ± 3.92^a^**	**9.74 ± 3.59^b^**	**17.68 ± 5.00^c^**	**23.18 ± 5.71^d^**	**16.63 ± 5.81^b,c^**	**28.48 ± 9.30^e^**
11	396.87	396.85	Ca II	11.70 ± 2.91^a^	7.94 ± 2.69^b^	13.39 ± 3.69^a^	17.90 ± 4.28^c^	12.59 ± 4.20^a^	21.25 ± 6.82^d^
12	422.68	422.67	Ca I	10.10 ± 3.16^a^	7.42 ± 2.08^b^	10.63 ± 3.04^a^	17.29 ± 3.45^c^	9.65 ± 2.18^a^	19.90 ± 3.19^d^
**13**	**589.03**	**589.00**	**Na I**	**12.86 ± 3.49^a^**	**3.15 ± 1.51^b^**	**6.86 ± 2.36^c^**	**4.89 ± 0.79^d^**	**26.90 ± 3.21^e^**	**62.00 ± 7.32^f^**
**14**	**589.60**	**589.59**	**Na I**	**8.67 ± 2.42^a^**	**2.26 ± 0.93^b^**	**4.38 ± 1.48^c^**	**3.28 ± 0.53^b,c^**	**18.08 ± 2.31^d^**	**44.06 ± 5.54^e^**
15	656.37	656.28	H	92.04 ± 20.31^a,b^	96.55 ± 19.04^b^	84.53 ± 17.31^a,c^	99.55 ± 12.95^b^	77.03 ± 19.51^c^	86.41 ± 16.56^a^
16	715.81	715.67	O I	8.72 ± 3.14^a,b^	8.67 ± 1.94^a,b^	8.13 ± 1.96^a,c^	10.08 ± 1.56^d^	7.49 ± 2.74^c^	9.37 ± 1.86^b,d^
17	742.49	742.36	N I	27.09 ± 9.86^a^	27.60 ± 6.34^a^	27.09 ± 6.48^a^	32.55 ± 4.76^b^	24.32 ± 8.94^a^	31.54 ± 6.09^b^
18	744.30	744.23	N I	55.62 ± 20.48^a^	56.49 ± 13.02^a^	55.69 ± 13.34^a^	66.12 ± 9.64^b^	49.59 ± 18.07^a^	66.20 ± 12.67^b^
19	746.92	746.83	N I	97.98 ± 36.16^a,b^	99.25 ± 22.13^b^	97.42 ± 23.14^a,b^	115.17 ± 16.63^c^	86.51 ± 31.62^a^	115.14 ± 22.01^c^
**20**	**766.57**	**766.49**	**K I**	**11.09 ± 3.25^a^**	**11.56 ± 1.82^a^**	**15.06 ± 1.74^b^**	**23.04 ± 3.59^c^**	**19.73 ± 2.73^d^**	**6.60 ± 1.14^e^**
**21**	**769.97**	**769.90**	**K I**	**8.67 ± 2.71^a^**	**9.19 ± 1.63^a^**	**12.11 ± 1.49^b^**	**18.71 ± 3.08^c^**	**15.93 ± 2.24^d^**	**5.17 ± 0.83^e^**
22	777.47	777.19	O I	247.36 ± 86.94^a^	256.27 ± 57.14^a,b^	251.38 ± 58.87^a^	282.16 ± 39.91^b^	217.06 ± 73.11^c^	284.15 ± 52.33^b^
23	818.57	818.49	N I	87.02 ± 32.60^a^	88.64 ± 20.21^a^	85.78 ± 20.64^a^	99.72 ± 14.36^b^	75.30 ± 27.25^c^	98.93 ± 19.18^b^
24	818.86	818.80	N I	100.44 ± 36.69^a,b^	100.40 ± 21.59^a,b^	94.75 ± 22.35^a,c^	111.12 ± 16.03^b^	83.57 ± 30.02^c^	111.82 ± 21.10^b^
25	820.15	820.04	N I	32.89 ± 12.77^a^	32.97 ± 7.34^a^	31.25 ± 7.63^a,b^	37.26 ± 5.29^c^	27.92 ± 10.19^b^	37.42 ± 7.27^c^
26	821.14	821.07	N I	58.82 ± 21.19^a,b^	56.65 ± 12.37^a,b^	53.41 ± 12.62^a,c^	63.39 ± 8.95^b,d^	47.67 ± 17.13^c^	63.93 ± 12.38^d^
27	821.73	821.63	N I	248.81 ± 86.24^a^	253.70 ± 53.64^a,b^	244.93 ± 57.61^a^	285.55 ± 41.05^c^	215.51 ± 77.86^d^	278.54 ± 51.68^b,c^
28	822.28	822.31	N I	54.39 ± 23.15^a^	53.31 ± 13.41^a^	52.52 ± 12.38^a^	62.73 ± 8.90^b^	47.79 ± 16.98^a^	70.90 ± 14.55^c^
29	822.43	Unknown	Unknown	60.74 ± 20.81^a^	59.80 ± 12.69^a^	57.26 ± 13.28^a^	70.97 ± 10.30^b^	53.47 ± 19.36^a^	68.50 ± 13.32^b^
30	824.36	824.24	N I	54.59 ± 19.07^a^	54.14 ± 11.57^a^	51.45 ± 11.82^a^	64.01 ± 9.25^b^	47.99 ± 17.54^a^	62.25 ± 11.95^b^
31	844.73	844.68	O I	183.54 ± 61.68^a,b^	182.26 ± 36.66^a,b^	169.94 ± 38.41^a,c^	198.32 ± 28.27^b^	153.31 ± 51.96^c^	201.58 ± 36.31^b^
32	856.86	856.77	N I	23.77 ± 8.77^a^	23.73 ± 5.17^a^	21.86 ± 5.12^a,b^	27.16 ± 4.05^c^	20.11 ± 7.24^b^	26.96 ± 5.20^c^
33	859.54	859.40	N I	34.89 ± 12.63^a,b^	32.32 ± 6.30^a^	28.32 ± 6.42^c^	36.00 ± 5.57^a,b^	27.24 ± 10.09^c^	37.10 ± 7.24^b^

* The values are expressed as mean ±SD (n = 40). Values marked by different superscript letters within a row are statistically different at the level p < 0.05. A1: acacia honey (Shaanxi); A2: acacia honey (Shanxi); A3: acacia honey (Jilin); M1: multi-floral honey (Shanxi); M2: multi-floral (Qinghai); M3: multi-floral (Hubei).

**Table 3:** Discriminant results of honey origins.

Sample	Model	Accuracy	Mean Average Precision
Mixture of acacia honey and multi-floral honey	LDA	84.1%	80.1%
	SVM	83.1%	79.3%
Acacia honey	LDA	74.1%	86.9%
	SVM	82.6%	89.5%
Multi-floral honey	LDA	98.6%	95.1%
	SVM	99.7%	99.7%
